# A non-synonymous single nucleotide polymorphism in *SIRT6* predicts neurological severity in Friedreich ataxia

**DOI:** 10.3389/fmolb.2022.933788

**Published:** 2022-09-05

**Authors:** Layne N. Rodden, Christian Rummey, Yi Na Dong, Sarah Lagedrost, Sean Regner, Alicia Brocht, Khalaf Bushara, Martin B. Delatycki, Christopher M. Gomez, Katherine Mathews, Sarah Murray, Susan Perlman, Bernard Ravina, S. H. Subramony, George Wilmot, Theresa Zesiewicz, Alessandra Bolotta, Alain Domissy, Christine Jespersen, Baohu Ji, Elisabetta Soragni, Joel M. Gottesfeld, David R. Lynch

**Affiliations:** ^1^ Departments of Pediatrics and Neurology, Children’s Hospital of Philadelphia, Perelman School of Medicine, University of Pennsylvania, Philadelphia, PA, United States; ^2^ Clinical Data Science GmbH, Basel, Switzerland; ^3^ University of Rochester, Rochester, NY, United States; ^4^ University of Minnesota, Minneapolis, MN, United States; ^5^ Murdoch Children’s Research Institute, Victorian Clinical Genetics Services, Melbourne, VIC, Australia; ^6^ Department of Neurology, The University of Chicago, Chicago, IL, United States; ^7^ Departments of Pediatrics and Neurology, University of Iowa Carver College of Medicine, Iowa City, IA, United States; ^8^ Department of Pathology, School of Medicine, University of California, San Diego, San Diego, CA, United States; ^9^ Department of Neurology, University of California, Los Angeles, Los Angeles, CA, United States; ^10^ Praxis Precision Medicines, Boston, MA, United States; ^11^ Department of Neurology, University of Florida, College of Medicine, Gainesville, FL, United States; ^12^ Department of Neurology, Emory University School of Medicine, Atlanta, GA, United States; ^13^ Department of Neurology, University of South Florida, Tampa, FL, United States; ^14^ The Scripps Research Institute, La Jolla, CA, United States

**Keywords:** ataxia, mitochondrion, modifier, clinical trial, mRNA profiling, SIRT6, epigenetic

## Abstract

**Introduction:** Friedreich ataxia (FRDA) is a recessive neurodegenerative disease characterized by progressive ataxia, dyscoordination, and loss of vision. The variable length of the pathogenic GAA triplet repeat expansion in the *FXN* gene in part explains the interindividual variability in the severity of disease. The GAA repeat expansion leads to epigenetic silencing of *FXN;* therefore, variability in properties of epigenetic effector proteins could also regulate the severity of FRDA.

**Methods:** In an exploratory analysis, DNA from 88 individuals with FRDA was analyzed to determine if any of five non-synonymous SNPs in *HDAC*s/*SIRT*s predicted FRDA disease severity. Results suggested the need for a full analysis at the rs352493 locus in *SIRT6* (*p*.Asn46Ser). In a cohort of 569 subjects with FRDA, disease features were compared between subjects homozygous for the common thymine *SIRT6* variant (TT) and those with the less common cytosine variant on one allele and thymine on the other (CT). The biochemical properties of both variants of SIRT6 were analyzed and compared.

**Results:** Linear regression in the exploratory cohort suggested that an SNP (rs352493) in *SIRT6* correlated with neurological severity in FRDA. The follow-up analysis in a larger cohort agreed with the initial result that the genotype of *SIRT6* at the locus rs352493 predicted the severity of disease features of FRDA. Those in the CT *SIRT6* group performed better on measures of neurological and visual function over time than those in the more common TT *SIRT6* group. The Asn to Ser amino acid change resulting from the SNP in *SIRT6* did not alter the expression or enzymatic activity of SIRT6 or frataxin, but iPSC-derived neurons from people with FRDA in the CT *SIRT6* group showed whole transcriptome differences compared to those in the TT *SIRT6* group.

**Conclusion:** People with FRDA in the CT *SIRT6* group have less severe neurological and visual dysfunction than those in the TT *SIRT6* group. Biochemical analyses indicate that the benefit conferred by T to C SNP in *SIRT6* does not come from altered expression or enzymatic activity of SIRT6 or frataxin but is associated with changes in the transcriptome.

## Introduction

Friedreich ataxia (FRDA) is an autosomal recessive multisystem disorder resulting from disease-causing variants of the *FXN* gene (Campuzano et al., 1996). Approximately 97% of individuals with FRDA have an expanded GAA triplet repeat in the first intron of both *FXN* alleles, while the remaining 3% carry an expanded GAA repeat on one allele and a point mutation or deletion on the other (Durr et al., 1996; Bidichandani et al., 1997; Forrest et al., 1998; Cossee et al., 1999; Zuhlke et al., 2004). The GAA repeat leads to transcriptional silencing via epigenetic mechanisms with consequent deficiency of the protein frataxin; most disease-causing point mutations lead to a relative lack of functional frataxin (Soragni et al., 2008; De Biase et al., 2009; Becker et al., 2017; Rodden et al., 2021). Frataxin deficiency ultimately leads to the features of FRDA, including ataxia, areflexia, loss of sensation and proprioception, dysarthria, and vision loss. Individuals with FRDA can also develop cardiomyopathy, scoliosis, diabetes mellitus, hypoacusis, and urinary dysfunction (Andermann et al., 1976; Geoffroy et al., 1976; Ackroyd et al., 1984; Durr et al., 1996; Filla et al., 1996; Lamont et al., 1997; Delatycki et al., 1999; McCabe et al., 2000).

Human frataxin is a highly conserved, 210 amino acid (∼25 kDa) protein encoded in the nucleus with an N-terminal mitochondrial targeting sequence (Koutnikova et al., 1998; Cavadini et al., 2000; Schmucker et al., 2008). Frataxin is ubiquitously, but differentially, expressed in all mitochondria-containing cell types. The exact function(s) of frataxin remains unclear, but it likely plays a major role in mitochondrial iron metabolism including iron binding, iron storage, iron–sulfur cluster (ISC) biosynthesis, and potential defense against reactive oxygen species (ROS) (Babcock et al., 1997; Gakh et al., 2002). Abnormalities in these processes secondarily influence the metabolic functions of mitochondria and might explain features of FRDA such as the elevated insulin resistance noted in most individuals with FRDA. Frataxin expression is under the control of metabolic pathways (such as the PPARγ pathway) and epigenetic mechanisms (histone acetylation and DNA methylation), and variations in these pathways may also influence the pathophysiology. The length of the shorter GAA repeat (GAA1) is the major genetic predictor of disease severity, as it correlates with frataxin expression and severity of clinical features. However, the variability in GAA repeat length can only explain ∼60% of the variability in age of onset (Durr et al., 1996; Filla et al., 1996; Lamont et al., 1997; Lynch et al., 2006; Reetz et al., 2015). Performance on clinical rating scales such as the Friedreich Ataxia Rating Scale (FARS) is similarly predicted by GAA1 repeat length and age, but *R* ^2^ values in such models have values of only 0.3–0.4 (Lynch et al., 2006). In addition, substantially different levels of severity of disease features have been found in sibships in which siblings have similar GAA1 lengths (Armani et al., 2006). Together, such results suggest that additional factors influence the severity of the disorder such as environmental events or other genetic influences.

Enzymes involved in protein acetylation regulate gene expression of many metabolic pathways and have specifically been implicated in FRDA (Herman et al., 2006; Soragni et al., 2012). Thus, these enzymes could regulate the features of FRDA, and polymorphisms in the genes coding for these proteins could modify the phenotype and severity of FRDA. In the present study, we have investigated this possibility by assessing the influence of polymorphisms in histone deacetylase (*HDAC*) and sirtuin (*SIRT*) genes on the severity of FRDA disease features, concentrating specifically on a non-synonymous SNP in Sirtuin6 (*SIRT6*).

## Results

### An analysis of potential disease modifiers points to SIRT6 as a candidate

In an exploratory evaluation, we examined the ability of a series of 47 non-synonymous SNPs in HDAC genes to predict phenotypic severity in FRDA. The HDAC genes in the original analysis were selected on the basis of containing known non-synonymous polymorphisms at the time of analysis in 1980. We first evaluated the prevalence of each SNP. The majority (42 SNPs) showed no allele variation in an initial sample of 138 subjects with FRDA, consistent with the restricted ethnic distribution of FRDA (Labuda et al., 2000), and the remaining five showed variability among those with FRDA. In 88 of the 138 individuals with FRDA on whom detailed clinical data were available, we used linear regression analysis to assess whether such polymorphisms contributed to the clinical severity of FRDA. Each individual SNP was included as an independent variable in models of a series of measures of FRDA disease severity, accounting for age, sex, and GAA repeat length. Of these five non-synonymous SNPs based on the *R* ^2^ of models and associated nominal *p* values (not corrected for multiple comparisons), a non-synonymous SNP in *SIRT6* was associated with differences in multiple neurological measures of FRDA (timed 25-foot walk [T25W], FARS score, and a composite score of timed upper limb and lower limb coordination and visual acuity [Z3]) (Supplementary Table S1). A repeat analysis in a larger cohort of 200 people with FRDA showed similar results (Table 1). Consequently, this SNP in *SIRT6* was further evaluated in a larger cohort of 536 people with FRDA.

**TABLE 1:** Associations of HDAC gene polymorphisms with parameters of FRDA disease progression in 200 individuals with FRDA. Values are *p* values for correlations. *p* values that are <0.05 are in bold. SNP = single nucleotide polymorphism, 9HPT = 9-hold peg test, T25FW = timed 25-foot walk, LCLA = low-contrast letter acuity, and FARS = Friedreich Ataxia Rating Scale. Z-score is calculated as (value - mean)/standard deviation. Z2 is the sum of the Z-scores for 9PHT and T25FW. Z3 is the sum of the Z-scores for 9HPT, T25FW, and LCLA.

SNP	Gene	Age of onset	Z2	Z3	9HPT	T25FW	FARS	LCLA
rs34402301	HDAC10	0.12	0.99	0.26	0.91	0.96	0.4	**0.04**
rs228757	HDAC5	0.92	0.79	0.96	0.89	0.56	0.35	0.57
rs1045288	SIRT3	0.24	0.66	0.37	0.34	0.67	0.4	0.42
rs34162626	SIRT5	0.69	0.88	0.34	0.94	0.35	0.79	0.15
rs352493	SIRT6	0.88	**0.0016**	**0.0008**	**0.0014**	**0.0064**	**0.011**	0.69

*p* values that are <0.05 are in bold.

### CT *SIRT6* genotype corresponds to less severe neurological dysfunction in Friedreich ataxia

We expanded the initial analysis by examining the *SIRT6* genotype in a total of 569 individuals with FRDA; 89% of patients (*n* = 511) were homozygous at the *SIRT6* SNP locus with thymine on both alleles (from here on, this group is known as “TT”), and 10% of individuals (*n* = 56) were heterozygous at the *SIRT6* SNP locus with cytosine on allele 1, and thiamine on allele 2 (from here on, this group is known as “CT”). The remaining individuals (<1%) (*n* = 2) were homozygous at the *SIRT6* SNP locus with cytosine on both alleles. Due to the small sample size, the two individuals homozygous for cytosine were not included in further analyses. In examining the demographics associated with each *SIRT6* genotype, the TT and CT sub-cohorts did not differ statistically in age, frataxin protein level, GAA1 length, or GAA2 length (the length of the longer GAA triplet repeat) (Table 2). Although the GAA1 length between these two groups is not statistically different, a difference of 90 GAA triplets may be biologically significant, so a third sub-cohort (TT_mod_) was created by randomly removing individuals from the TT sub-cohort with GAA1 lengths larger than the median until the median GAA1 length was within ten triplets of those in the CT group. Those in the CT group had a later age of onset than those in the TT group (14 ± 10 years vs. 11 ± 8 years, Mann–Whitney test; *p* = 0.02), but this statistical difference did not persist when compared to those in the TT_mod_ group (14 ± 10 years vs. 12 ± 9 years, Mann–Whitney test; *p* = 0.1).

**TABLE 2:** Patient demographics. TT_mod_ was created to account for the difference in GAA1 length between CT and TT by randomly removing subjects in the TT group with GAA1 length above the median until the GAA1 of TT_mod_ was within 10 triplets of CT. Age and age of onset (AoO) are reported in years. Frataxin protein levels were reported as % of non-FRDA healthy controls (controls consist of a collection of 5–10 people who do not have FRDA and are not carriers of the GAA-TR expansion. These controls were sampled repeatedly and run alongside patient samples in the same assay). GAA1 and GAA2 were reported as the number of triplets. Values are reported at median±standard deviation with the range of values in parentheses. Medians were compared with the Mann–Whitney test. *AoO showed *p* = 0.02 for CT vs. TT, all other variables were not significant.

	CT *n* = 56	TT *n* = 480	TT_mod_ *n* = 350
**Female, %**	59	50	50
**Age, median (range)**	27.4 ± 15.8 (9–78)	26.2 ± 14.1 (8–85)	26.2 ± 15.3 (8–85)
^*^ **AoO, median (range)**	14 ± 10 (2–43)	11 ± 8 (1–63)	12 ± 9 (1–63)
**Frataxin protein, median (range)**	30.8 ± 17.4 (3.6–71.5)	20.7 ± 15.5 (1.5–109.3)	22.6 ± 16.2 (2.1–109.3)
**GAA1, median (range)**	600 ± 260 (41–1,000)	690 ± 223 (42–1,150)	603 ± 235 (42–1,150)
**GAA2, median (range)**	900 ± 199 (450–1,300)	932 ± 218 (99–1,555)	931 ± 233 (99–1,555)

**FIGURE 1:**
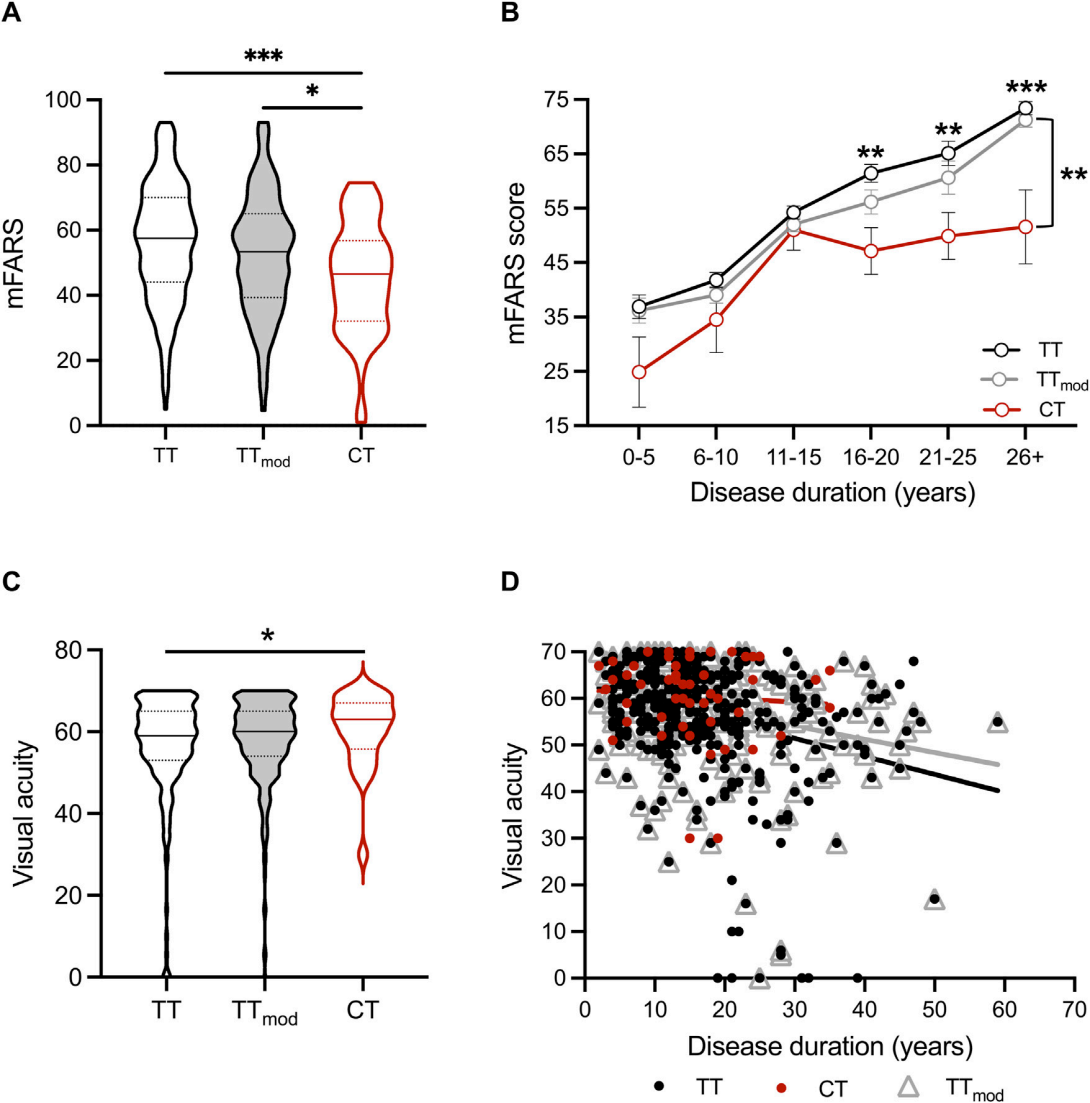
People with FRDA with the CT *SIRT6* genotype have better neurological scores, including vision, than TT and TT_mod_ *SIRT6* genotypes. **(A)** Modified Friedreich Ataxia Rating Scale (mFARS) scores and **(C)** visual acuity scores for all sub-cohorts displayed as violin plots (solid lines indicate medians, and dotted lines indicate quartiles). Medians were compared using the Kruskal–Wallis test. **(B)** Mean ± SEM mFARS scores plotted over disease duration in years for all sub-cohorts. Means were compared at each disease duration range time point for CT and TT_mod_ groups with two-way ANOVA (*p* < 0.0001) and Sidak’s posthoc analysis (on graph: asterisks above the TT line = TT vs. CT, and asterisks to the right of bracket = TT_mod_ vs. CT). **(D)** Visual acuity plotted over disease duration in years for all sub-cohorts (CT: *R* ^2^ = 0.006, *p* = *n*.s., TT: *R* ^2^ = 0.09, *p* < 0.0001, TTmod: *R* ^2^ = 0.08, *p* < 0.0001). * = *p* < 0.05, ** = *p* < 0.01, *** = *p* < 0.001, and *** = 0 < 0.0001.

To determine if the genotype of *SIRT6* is related to the severity of clinical features of FRDA, we analyzed modified Friedreich Ataxia Rating Scale (mFARS) scores, a composite measure of neurological dysfunction in FRDA. Higher mFARS scores reflect more severe neurological dysfunction, and the average increase in mFARS scores in all FRDA patients is ∼1.5 points/year (Patel et al., 2016). The CT cohort had lower mFARS scores overall and throughout disease duration than both the TT and TT_mod_ cohorts (Figures 1A, B). In addition, mFARS scores in the CT cohort plateaued after roughly 15 years of disease, while TT and TT_mod_ mFARS scores continued to increase. The difference of 21 mFARS points between the CT group (52 ± 7 points) and the TT group (73 ± 1 mFARS points) at 26 + years of disease equates to fewer years of disease duration (14 years) for the CT group over the same period of time.

In a multivariable linear regression model, GAA1 length, age, and sex collectively predicted mFARS scores in FRDA (*R* ^2^ = 0.38, *p* < 0.0001). Adding the *SIRT6* genotype increased the overall *R* ^2^ value (*R* ^2^ = 0.40) as well as being individually significant, thus improving the prediction of the mFARS score (Table 3). Other measures of neurological dysfunction in FRDA, 9-hole peg test (9HPT), timed 25-foot walk (T25FW), and the combination “Z2 score,” were also predicted by GAA1 length and age [9HPT: *R* ^2^ = 0.27; T25FW: *R* ^2^ = 0.24; Z2: *R* ^2^ = 0.30]; again, adding *SIRT6* genotype to a multivariable regression analysis improved the prediction of all three measures [9HPT: *R* ^2^ = 0.31; T25FW: *R* ^2^ = 0.29; Z2: *R* ^2^ = 0.34] (Table 3). Similar results were seen with the TT_mod_ sub-cohort (data not shown). Thus, the *SIRT6* SNP genotype predicts neurological outcomes in FRDA, and the CT genotype is associated with better neurological function over time.

**TABLE 3:** *SIRT6* genotype significantly improves predictions of clinical outcomes measures in FRDA. Column “comparison variables” lists independent variables used in multivariable comparisons. Overall, *R* ^2^ and *p* values as well as *p* values for each individual variable in the statistical model are shown. mFARS = modified Friedreich ataxia rating scale, 9HPT Z-score = 9-hole peg test Z-score, T25FW Z-score = timed 25-foot walk Z-score. Z scores were calculated as follows: (value - mean)/standard deviation. Z2 is the sum of the Z scores for 9HPT and T25FW.

Comparison variables	n	*R* ^2^	Overall *p*	Individual *p*
**mFARS**				
Age, GAA1, and sex	531	0.38	<0.0001	Age < 0.0001
				GAA1 < 0.0001
				Sex = *n*.s.
Age, GAA1, sex, and SIRT6	531	0.4	<0.0001	Age < 0.0001
				GAA1 < 0.0001
				Sex = *n*.s.
				SIRT6 < 0.0001
**9HPT Z-score**				
Age, GAA1, and sex	531	0.27	<0.0001	Age < 0.0001
				GAA1 < 0.0001
				Sex = 0.009
Age, GAA1, sex, and SIRT6	531	0.31	<0.0001	Age < 0.0001
				GAA1 < 0.0001
				Sex = 0.005
				SIRT6 < 0.0001
**T25FW Z-score**				
Age, GAA1, and sex	531	0.24	<0.0001	Age < 0.0001
				GAA1 < 0.0001
				Sex = *n*.s.
Age, GAA1, sex, and SIRT6	531	0.29	<0.0001	Age < 0.0001
				GAA1 < 0.0001
				Sex = *n*.s.
				SIRT6 < 0.0001
**Z2 (9HPT + T25FW)**				
Age, GAA1, and sex	531	0.3	<0.0001	Age < 0.0001
				GAA1 < 0.0001
				Sex = 0.01
Age, GAA1, sex, and SIRT6	531	0.34	<0.0001	Age < 0.0001
				GAA1 < 0.0001
				Sex = 0.03
				SIRT6 < 0.0001

### CT *SIRT6* genotype protects against severe vision loss in Friedreich ataxia

In visual acuity testing with Early Treatment Diabetic Retinopathy Study (ETDRS) eye charts, individuals with the CT genotype had better visual acuity than individuals with TT (*p* = 0.03) (Figure 1C). When measured throughout disease duration, visual acuity in TT and TT_mod_ cohorts worsened over time (TT: *R* ^2^ = 0.09, *p* < 0.0001, TTmod: *R* ^2^ = 0.08, *p* < 0.0001), whereas the visual acuity of those with CT did not change over time (CT: *R* ^2^ = 0.006, *p* = *n*.s.) (Figure 1D).

### *SIRT6* genotype does not predict prevalence of cardiomyopathy, scoliosis, or diabetes in Friedreich ataxia

**FIGURE 2:**
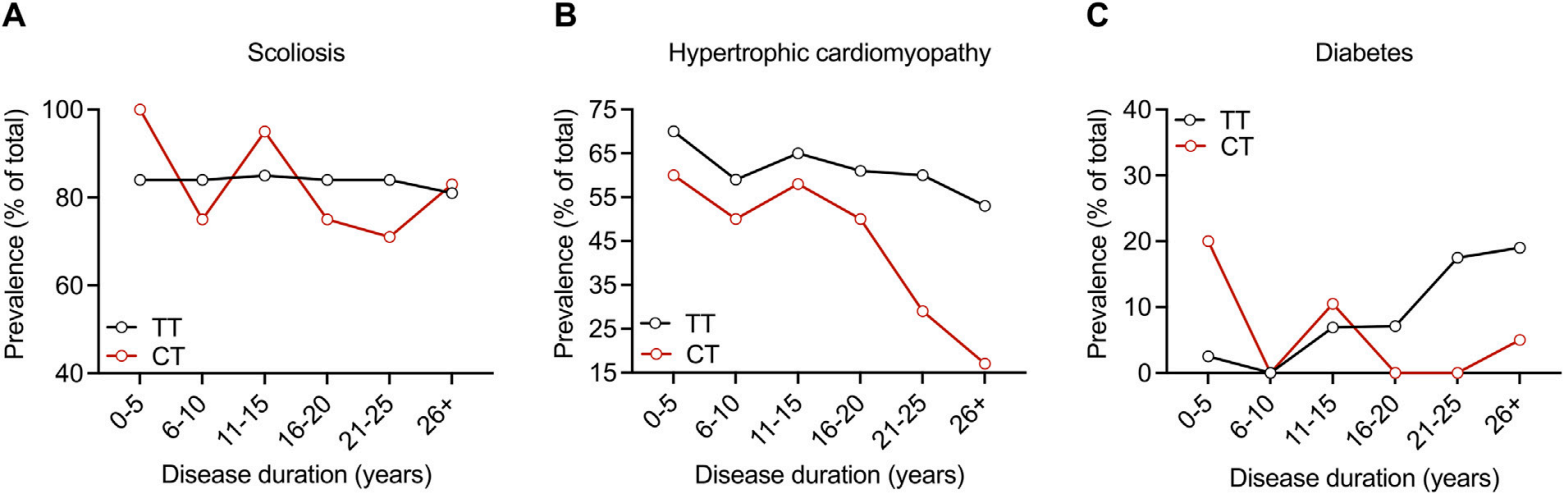
*SIRT6* genotype does not influence the prevalence of scoliosis, hypertrophic cardiomyopathy, or diabetes throughout disease duration in FRDA. Prevalence of **(A)** scoliosis, **(B)** hypertrophic cardiomyopathy, and **(C)** diabetes plotted as % of the total over ranges of disease duration for both sub-cohorts. Proportions were compared at each disease duration range time point with Fisher’s exact test. All comparisons showed no statistical difference.

In the entire cohort, 84, 59.3, and 8.6% had scoliosis, hypertrophic cardiomyopathy, and diabetes, respectively. The prevalence of all three of these features was similar in both the TT and CT sub-cohorts, and we found no statistical differences between the groups (Table 4). We analyzed these disease features over time and found a lower prevalence of hypertrophic cardiomyopathy and diabetes in the CT group at later disease durations; however, Fisher’s exact test found no statistically significant differences at any point in disease duration for these features (Figures 2A–C). The modifier effect of the *SIRT6* SNP on FRDA disease features seems to be exclusive to neurological features of the disease.

**TABLE 4:** SIRT6 genotype does not influence the prevalence of scoliosis, hypertrophic cardiomyopathy, or diabetes in FRDA. Prevalence of scoliosis, hypertrophic cardiomyopathy (HCMP), and diabetes mellitus (DM) in the total cohort and both sub-cohorts TT and CT. Fisher’s exact test was used to statically analyze each feature between TT and CT sub-groups. All comparisons showed no statistical difference.

Feature	Total cohort	TT	CT
**Scoliosis (% of total)**	84.0	83.9	84.9
**HCMP (% of total)**	59.3	60.8	47.1
**DM (% of total)**	8.6	8.7	7.6

### *SIRT6* genotype does not alter the biochemical properties of *SIRT6* or differentially affect expression of *FXN*

The cytosine SNP in *SIRT6* encodes for the amino acid serine at position 46 (S46) in the SIRT6 protein, and the thiamine encodes for the amino acid asparagine at position 46 (N46) in the SIRT6 protein. A previous study found no difference in biochemical properties or enzymatic activity of S46 vs. N46 SIRT6 (Kugel et al., 2016), and structural prediction software (www.sift.jcvi.org) suggests that the amino acid change does not impact the SIRT6 protein structure. We tested the possibility that the CT genotype confers improved clinical outcomes in individuals with FRDA by positively affecting the expression of *FXN*. We measured *FXN* mRNA levels in blood samples collected from the 138 subjects of our exploratory analysis as well as the larger cohort of 536 subjects and found no correlation between SNP status and *FXN* mRNA levels in multivariable regression models with GAA1, a known predictor of frataxin levels (data not shown). Similar results were found using a collection of FRDA fibroblast lines. We derived iPSC lines from these fibroblasts, which were fully characterized for pluripotency gene and protein expression (Supplementary Figures S1, S2) and derived neuronal cells from the iPSCs (Soragni et al., 2014). We confirmed the SNP status of the neuronal cells by sequencing PCR products (Supplementary Figure S3A). We found no correlation between *SIRT6* SNP status and *FXN* mRNA levels in iPSCs or derived neuronal cells (Supplementary Figure S3B). Moreover, shRNA silencing of either *SIRT1* or *SIRT6* had no effect on *FXN* mRNA levels in FRDA neuronal cells (Supplementary Figures S3C,D). Based on these findings, it is unlikely that the *SIRT6* SNPs differentially affect *FXN* gene expression. *SIRT6* mRNA and protein levels were also similar in FRDA fibroblasts (Supplementary Figures S4A, B), and SIRT6 protein levels were similar in neuronal cells from both genotypes (Supplementary Figure S4C). Since Western blots do not discriminate between N46 SIRT6 and S46 SIRT6, we expressed these protein isoforms in HEK293 cells as FLAG-tagged constructs in the pcDNA3.1 vector and probed Western blots of both crude protein lysates and FLAG-purified proteins with SIRT6 antibody, but again we found no differences in expression or stability of N46 SIRT6 versus S46 SIRT6 protein (Supplementary Figure S5). Potential phosphorylation at amino acid S46 was monitored by mass spectrometry of FLAG-tagged S46 SIRT6 expressed HEK293 cells, but no tryptic peptides corresponding to S46p were identified (not shown). We previously found that *FXN* GAA repeats expand during the propagation of iPSCs (Pan et al., 2011; Soragni et al., 2014) in a manner analogous to intergenerational repeat expansion in the maternal germ line. We found that the GAA repeat expansion is comparable during the propagation of iPSCs from individuals with FRDA with both TT and CT SNP genotypes (Supplementary Figure S6). Lastly, knockdown of SIRT6 in GM03816 TT iPSCs does not affect repeat expansion (data not shown), suggesting that the SIRT6 genotype is not involved in GAA repeat expansion, at least in this iPS cell model.

### Individuals with Friedreich ataxia with CT *SIRT6* genotype have altered transcriptomes

Since we found no differences in the biochemical properties of SIRT6 and no difference in the expression level of *FXN* based on the SIRT6 genotype, we hypothesized that mechanisms unrelated to differences in protein properties are responsible for the less severe neurological phenotype in people with the CT *SIRT6* genotype. We compared global gene expression profiles of FRDA iPSC-derived neuronal cells that harbor the SIRT6 TT genotype (from lines GM04078 and GM03816) to similar cells that harbor the SIRT6 CT (lines FA71 and FA272) by using unbiased RNA sequencing (RNA-seq) technique. Principal component analysis (PCA) shows clustering dependent on both GAA repeat lengths and *SIRT6* genotype (Supplementary Figure S7). One thousand two hundred and eighty-six genes were found to be differentially expressed (DE) between CT and TT lines (with a false discovery rate (FDR) < 0.01, *p*-value of 0.05, and logFC of at least 0.6 up or downregulated), of which 730 were upregulated and 556 were downregulated. Top enriched pathways for these DE genes, identified within the KEGG Pathway database (Release 96.0+/11–21, November 20), are reported in Supplementary Table S2.

We next performed chromatin immunoprecipitation sequencing (ChIP-seq) using the same neuronal cells and an antibody to SIRT6. Using MACS2 (version v2.2.7.10) software, 2,155 peaks were called that were differentially enriched in the CT neuronal cells than the TT cells. One hundred and seventy-eight peaks were found to be localized in promoter elements and transcription start sites (data not shown). Amongst the SIRT6-occupied genes, five were also found to be differentially expressed in the RNA-seq data. These are ETV7 (ETS variant transcription factor 7), IGF2BP2 (insulin-like growth factor 2 mRNA-binding protein 2), KHSRP (KH-type splicing regulatory protein), SEC61A (transport protein Sec61 subunit alpha isoform 2), and SUGP2 (SURP and G-patch domain-containing protein 2) (Table 5). Notably, four of these genes encode proteins involved in transcription and RNA processing, perhaps providing a clue as to the molecular basis for the clinical observations in people with CT *SIRT6*.

**TABLE 5:** Fold changes (FC) and *p* values for the five genes found to be differentially expressed and differentially occupied by SIRT6 in iPSC-neurons from people with FRDA with the CT SIRT6 genotype vs. the TT SIRT6 genotype.

Symbol	Log FC	FC (CT vs. TT)	*p*-value
SUGP2	0.475	1.39	6.757e-6
ETV7	1.126	2.18	0.022
IGF2BP2	-2.531	0.17	3.539e-5
SEC61A2	0.628	1.55	2.145e-4
KHSRP	-0.477	0.72	0.003

We queried a publicly available transcriptomic data set (Nachun et al., 2018) generated from blood from people with FRDA for differential gene expression profiles between a larger number of patients with CT *SIRT6* and TT *SIRT6*. Nine hundred and twenty genes were differentially expressed in the CT group compared to the TT group, and 832 genes were differentially expressed in the CT group compared to the TT_mod_ group. Six hundred and sixty-four differentially expressed genes overlapped between these two sets and were used for the subsequent analyses. Genes were filtered by fold change, and the top differentially expressed genes that are known to be involved in a pathway(s) relevant to FRDA and SIRT6 function including chromatin organization, DNA damage repair, metabolism, retinol processing, ATP synthesis, and others are listed in Supplementary Table S3. Although these findings are statistically significant, the small fold-changes between the two genotypes (1–10%) in blood and fibroblasts are pathophysiologically unconvincing.

**FIGURE 3:**
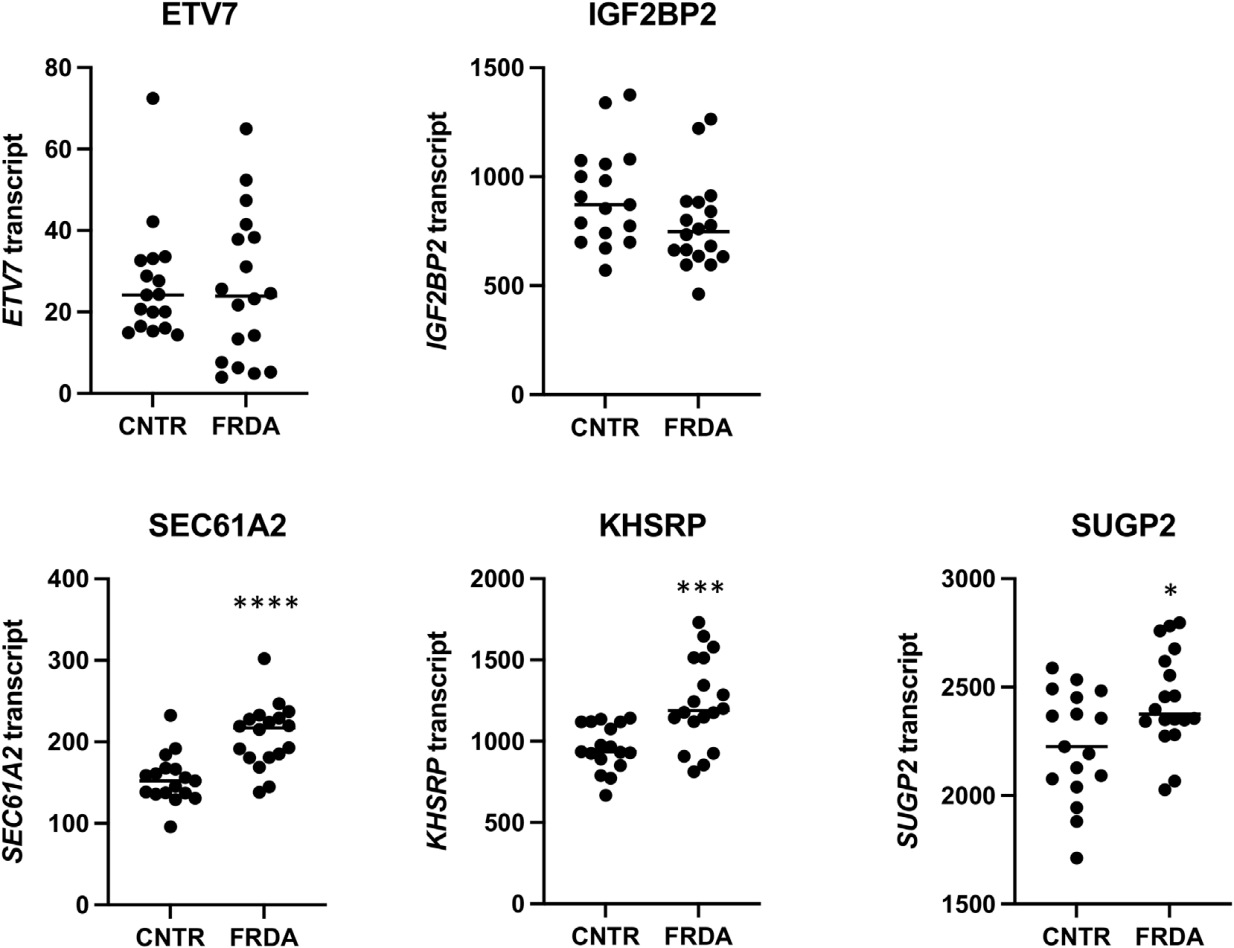
Relative mRNA values from a publicly available RNA-seq data set for the five DE genes found in the iPSC-neuron analysis (ETV7, IGF2BP2, SEC61A2, KHSRP, and SUGP2) shown in non-FRDA control (CNTR) fibroblasts and FRDA fibroblasts. * = *p* < 0.05, *** = *p* < 0.001, and **** = *p* < 0.0001.

A second publicly available transcriptomic data set (Napierala et al., 2017) was analyzed for DE genes in fibroblasts from 18 people with FRDA (and 17 non-FRDA controls). Since only one person with FRDA out of the 18 in this study has the CT *SIRT6* variant, we were unable to analyze DE genes in CT vs. TT groups. Instead, we looked at the 5 DE and differentially occupied genes identified in the iPSC-neuron analysis in FRDA vs. non-FRDA controls and the hypothesis that the SIRT6 CT genotype results in a transcriptomic signature that compensates for the changes in the FRDA transcriptome (Figure 3). SEC61A2, KHSRP, and SUGP2 were found to be significantly upregulated in FRDA, and KHSRP changed in the opposite direction in CT neurons vs. TT neurons, which might indicate that CT SIRT6 genotype results in a compensatory mechanism to “normalize” expression of this disrupted gene in FRDA.

## Discussion

The present study demonstrates that a non-synonymous SNP in *SIRT6* is associated with less severe neurological dysfunction in FRDA. The *SIRT6* genotype improves predictions of disease features in those with FRDA by a modest (2–5%) but significant amount. Those with the less common *SIRT6* genotype, CT, have better neurological outcomes as well as less incidence of severe vision loss throughout the course of disease. This effect persists when differences in GAA repeat length are accounted for statistically or by random matching of the sub-cohort.

There is a neurological selectivity to the effects of CT *SIRT6* genotype. Those in the CT group showed a lower prevalence of cardiomyopathy and diabetes at later disease durations, but the change was not statistically significant. This could be because there is indeed no difference between groups or perhaps due to the inability of Fisher’s exact test to determine significance at lower effect sizes. The effect of the genotype on neurological function was statistically greatest in individuals with longer disease durations. The effect was statistically minimal in affecting the age of onset but had higher levels of significance in predictions of change over time. In addition, in exploratory data, no other non-synonymous SNP had a meaningful effect on any aspect of FRDA, a finding that directed us to assess the *SIRT 6* genotype in great detail.

While the statistical association of the CT *SIRT6* genotype with less severe neurologic dysfunction was seen by multiple types of clinical measures, the mechanism is not clear. As SIRT6 is a protein deacetylase that modulates transcription, SIRT6 could act directly at the *FXN* locus to modify its transcription. Since frataxin expression did not differ between CT and TT groups, and the ChIP-Seq analysis in iPSC-derived neurons did not implicate differential occupancy of SIRT6 at the *FXN* locus based on *SIRT6* genotype, this possibility is not supported by our data. As frataxin deficiency is the root cause of FRDA, many consequent downstream pathways are altered in patients as well, either as a compensatory mechanism or as a direct consequence of frataxin deficiency. As we saw no difference in average or median frataxin levels between the two groups of patients or in *FXN* transcript levels in cell culture studies, it seems likely that the mechanism(s) associated with the CT *SIRT6* genotype is something other than direct modulation of frataxin.

Transcriptional regulation *via* SIRT6 affects many genes involved in downstream pathways such as glucose metabolism, DNA damage repair, aging, and insulin signaling. It is plausible that the N to S amino acid change in SIRT6 changes the enzymatic properties of SIRT6, which could modulate downstream pathways differently between the two genotypes. This possibility, however, is also not supported by our data or others’ (Patel et al., 2016); others have found no difference in enzymatic activity, and we found no difference in expression, albeit with small sample sizes, between the two SIRT6 variants. We cannot rule out the possibility of a subtle effect on SIRT6 activity by the presence of N to S amino acid change that may compound over time or a difference in regulation or localization of SIRT6 activity in a specific situation. The location of the N--> S amino acid is outside of the catalytic domain of the enzyme but is within the Rossmann fold involved in NAD+ binding (Pan et al., 2011). Another possibility is that the N to S SNP changes the interacting proteins or molecules of SIRT6, a mechanism that has not been explored.

The differential transcriptome expression and ChIP-seq occupancy data from iPSC-derived neurons show that the CT genotype of *SIRT6* provides some indication of differences in downstream pathways that may be relevant to FRDA, but they do not identify a detailed mechanism. These findings were not replicated in publicly available RNA-seq data from patient-derived blood samples. Blood is a non-neurological tissue and is unaffected clinically in FRDA, so this discrepancy may further support the selective effect that the CT *SIRT6* genotype provided only to the neurological symptoms of FRDA. Another possible explanation for the discrepancy is that the data set used in this study showed small/subtle changes between FRDA and control samples in the original analysis (Nachun et al., 2018); therefore, we might not expect to see robust changes between the CT and TT groups of FRDA patients, which are more similar to each other than controls vs. all FRDA patients. Other publicly available data with FRDA samples exist, but the *SIRT6* SNP status is either undeterminable (the samples are not in our database), or they do not contain enough CT FRDA patients. One example is GSE104288, RNA-seq data from a collection of fibroblasts, which was queried in this study. Out of the 18 FRDA fibroblasts in this collection, only one had the CT *SIRT6* genotype. We were, therefore, limited to analyzing controls vs. FRDA patients and comparing those results with the iPSC-neuron results. One DE gene identified from the iPSC-neuron analysis was found to change in the opposite direction in controls vs. FRDA in fibroblasts, KHSRP. The functions of KHSRP include regulation of gene expression in response to endoplasmic reticulum stress, metabolism of RNA, translational control, and regulation of mRNA stability. It is also implicated in the pathophysiology of spinal muscular atrophy. Moreover, this result is interesting, and we are cautious to overinterpret the less severe neurological severity in the CT SIRT6 group as being the result of changes in one downstream gene/pathway. We suggest future studies expand on the RNA-seq analysis in relevant cell types/tissues with more CT and TT samples.

A final possibility is that the SNP in *SIRT6* is not the true modifier but simply in linkage disequilibrium with another polymorphism. This seems unlikely since assessment of other SNPs in this region demonstrated lesser effects on the features of FRDA. Still, until a direct biochemical or genetic mechanism is found, linkage disequilibrium remains a possibility.

In the present study, we have identified a non-synonymous SNP in *SIRT6* as a potential modifier of FRDA neurological features. The less common CT genotype of *SIRT6* is linked to better neurological and visual health in patients. Future studies should focus on specific pathways implicated in the transcriptomic data to determine if the mechanism(s) of this potential modifier is a novel therapeutic target for FRDA.

## Methods

### Subjects, cell lines, and data sets

#### Friedreich ataxia clinical outcome measures study cohort

All protocols were approved by the Institutional Review Board at the Children’s Hospital of Philadelphia and other sites. Informed consent was obtained before participation. Genetic confirmation with GAA repeat length determination was obtained on all subjects *via* commercial or research testing. Subjects participating in the Friedreich Ataxia Clinical Outcome Measures Study (FACOMS) were evaluated at one of 12 sites (Friedman et al., 2010; Patel et al., 2016; Rummey et al., 2020; Xiong et al., 2020). Data collected as part of this study included medical history and several quantitative measures of neurological or visual function: 1. The Friedreich Ataxia Rating Scale (FARS) and modified FARS (mFARS), a quantified neurological exam used in the evaluation of FRDA, 2. timed 25-foot walk (T25FW), 3. 9-hole peg test (9HPT), and 4. visual function testing using ETDRS vision charts. For this experiment, only high-contrast visual acuity charts were used, leading to a maximum score of 70. The visual acuity, T25W, 9HPT, and FARS/mFARS were performed and scored according to protocols described previously (Friedman et al., 2010; Patel et al., 2016; Rummey et al., 2020; Xiong et al., 2020). Two performance measure composites designated Z2 and Z3 were calculated from the performance measures (visual acuity, T25W, and 9HPT) as described previously (Lynch et al., 2006).

#### Data sets for transcriptome analysis and comparison

We used a publicly available data set for transcriptome analysis in a larger number of CT and TT samples. The data set was generated *via* expression profiling by an array of 733 blood samples from people with FRDA; 56, 480, and 350 of which are in the CT group, TT group, and TT_mod_ group, respectively (GEO accession# GSE102008), and analyzed via GEO2R software for differentially expressed genes between CT and TT patients (Nachun et al., 2018).

A data set generated by RNA-seq of 18 FRDA and 17 non-FRDA control fibroblast samples was used to determine differentially expressed genes between FRDA and non-FDRA fibroblasts by obtaining raw expression values for each subject (GEO accession # GSE104288) (Napierala et al., 2017).

#### Cell lines

Fibroblasts, human-induced pluripotent stem cells (hiPSCs), neurospheres, neurons, and HEK293T cells were grown at 37°C and 5% CO_2_. Fibroblasts were cultured with 10% FBS in minimal essential medium, 2 mM glutamine, 1% non-essential amino acids, 20 mM HEPES, and 1% antibiotic/antimycotic (Invitrogen). iPSCs were grown on irradiated mouse embryonic fibroblasts (GlobalStem, Rockville, MD) in DMEM/F-12 with 20% KnockOut serum replacement, 1 mM glutamine, 1% nonessential amino acids, 1% antibiotic/antimycotic, 0.1 mM beta-mercaptoethanol (Invitrogen), and 20 ng/ml basic FGF (Stemgent, San Diego, CA) and passaged manually every 7 or 8 days. Neurospheres were grown in Neurobasal-A medium with 2% B-27 supplement, 1% N-2 supplement, 2 mM glutamine, 1% antibiotic/antimycotic, 10 mM HEPES, 20 ng/ml basic FGF, and 20 ng/ml EGF (R&D Systems). Neuronal cells were grown on Matrigel in Neurobasal-A medium with 2% B-27 supplement, 1% N-2 supplement, 2 mM glutamine, 1% antibiotic/antimycotic, and 10 mM HEPES. HEK293T cells were grown with 10% FBS in DMEM, 2 mM glutamine, 20 mM HEPES, 1% nonessential amino acids, and 1% antibiotic/antimycotic.

To isolate primary fibroblasts, dermal explant cultures were established from dispase-treated skin biopsies on fibronectin underneath a glass coverslip with fibroblast media after 5–7 days. After establishment, primary dermal fibroblasts were cultured as described earlier. Biopsies were performed under approved Human Subjects Protocols at Children’s Hospital of Philadelphia and Scripps.

### Derivation of iPSCs and neuronal differentiation

iPSC line generation and characterization were described previously (Ku et al., 2010). Neuronal differentiation was performed as described (Lai et al., 2019). Briefly, iPSCs grown on Matrigel (Corning, Coming, NY) and mTeSR (Stem Cell Technologies, Vancouver, Canada) were treated for 10 days in E6 medium (Stem Cell Technologies, Vancouver, Canada) with 0.5 μM LDN-193189, 10 μM SB431542, and 20 μg/ml FGF2. iPSC colonies were dissociated with Accutase (Innovative Cell Technologies, San Diego, CA) and plated in AggreWell plates (Stem Cell Technologies, Vancouver, Canada) at a density of 1,000 cells per microwell. Neurospheres were grown in suspension for 1 week in Neurobasal-A medium (Thermo Fisher Scientific, Waltham, MA), supplemented with N2 and B27 supplements (Thermo Fisher Scientific, Waltham, MA) and FGF2 and EGF, both at 20 μg/ml. Neurospheres were then plated on Matrigel and grown in the same medium as mentioned earlier until rosettes appeared. Rosettes were manually isolated, grown for 4–7 days in suspension, then dissociated with Accutase, and plated on Matrigel at 200,000 cells/cm2. To induce neuronal differentiation, cells were grown in the same medium as mentioned earlier without FGF2 and EGF for 14 days.

### Genotyping and exploratory analysis of histone deacetylase SNPs

Genomic DNA from 138 subjects with FRDA from multiple sites in the United States was genotyped at 47 non-synonymous SNPs in HDAC genes (*HDAC10*, *HDAC5*, *SIRT3*, *SIRT5*, and *SIRT6*), and SNP scores were assigned based on mutation status. Following preliminary analysis, further genotyping was performed and included subjects from all countries in FACOMS. SNP array data (Illumina Infinium™ OmniExpressExome-8 v1.6 array) were collected from 632 subjects of the FACOMS cohort. Genotyping was performed at the Broad Institute. SNPs, samples, and genotypes were filtered based on standard quality control metrics (Turner et al., 2011) and concordance with de-identified clinical data using PLINK 1.9 (Chang et al., 2015). Genotypes from 569 subjects for rs352493 passed quality control filters.

### Immunostaining

Cells were fixed in 4% paraformaldehyde and permeabilized with PBS 1X and 0.1% Triton X-100. Primary antibodies to Oct4, Tra1-81, Tra1-61, and SSEA4 were obtained from Millipore (MAB4305, MAB4381, MAB4303, and MAB4304) and were all used at 1:50 dilution and incubated at 4°C overnight. Fluorescent secondary antibodies were obtained from Santa Cruz Biotechnology (anti-mouse Texas Red conjugate, anti-mouse FITC conjugate, anti-rat 8 Texas Red conjugate, and anti-rat FITC conjugate). All secondary antibodies were used at 1:100 dilutions and incubated at room temperature for 1 h, followed by nuclear staining with DAPI.

### Transfection of HEK293 cells with FLAG-tagged SIRT6

HEK293T cells were seeded at a density of 0.3 × 10^6^/ml in DMEM medium without antibiotics and transfected with the carrier pcDNA3.1 or FLAG-tagged fusion proteins cloned in pcDNA3.1 (Addegene). The CT version of *SIRT6* was derived by site-directed mutagenesis of the TT version available from Addgene, which is cloned in the FLAG-tagged mammalian expression vector pcDNA3.1 (http://www.addgene.org/13817/). The identity of the two constructs was confirmed by DNA sequencing. Transfection of HEK293 cells was performed using Lipofectamine (Life Technologies) as described by the supplier.

### Polymerase chain reaction and quantitative reverse transcription-polymerase chain reaction

Total RNA was purified with the RNeasy Plus Mini kit (QIAGEN) according to the manufacturer’s instruction. Genomic DNA was purified by isopropanol precipitation of cell lysates prepared in total cell lysis buffer (100 mM Tris, 5 mM EDTA, 0.2% SDS, 0.2 M NaCl, and 200 mg/ml proteinase K [pH 8]). For GAA triplet repeat length conventional PCR, Phusion polymerase (New England Biolabs, Ipswich, MA) was used according to the manufacturer’s instruction; 20 ng of DNA and 0.1 μM primers GAA-104F and GAA-629R (Ku et al., 2010) were used in 20 μL of reactions cycled through the following conditions: denaturation at 98°C for 5 s, annealing at 70°C for 15 s, and extension at 72°C for 90 s for 40 cycles with a 5-min initial denaturation and a 5-min final extension. Quantitation of PCR band size was performed using an inverse power function directly correlating gel migration of a molecular weight ladder to its known size. Quantitative RT-PCR analysis was performed with the qScript One-Step SYBR Green qRT-PCR Kit (Quanta Biosciences) according to the manufacturer’s instruction. Analysis of relative qRT-PCR data was performed via the 2−ΔΔCT method and normalized to GAPDH mRNA levels.

### Western blot analysis

Whole-cell extracts (in 50 mM Tris [pH 7.4], 150 mM NaCl, 10% glycerol, 0.5% Triton X-100, and protease inhibitor; Roche) were electrophoresed in polyacrylamide gels and transferred onto nitrocellulose membranes. Primary antibodies were incubated overnight, and secondary antibodies were incubated for 1 h at room temperature. Antibody incubation was performed in 3% BSA in TBS containing 0.1% Tween 20. Anti-α LN 13 (2765S) and anti-GAPDH (9,484) antibodies were obtained from Cell Signaling and used at 1:2,000 and 1:1,000 dilutions, respectively. Anti-SIRT6 (2590S) and anti-SIRT6 (62,739) antibodies were obtained from Cell Signaling and Abcam, respectively, and used at 1:250 dilutions. IRDye 680LT-conjugated goat anti-mouse IgG (H + L; 926–68020) and IRDye 800CW-conjugated goat anti-rabbit IgG (H + L; 926–32211) secondary antibodies were obtained from LI-COR Bioscience and used at a dilution of 1:5,000.

### Ribonucleic acid sequencing

RNA was isolated using the RNeasy mini kit (QIAGEN, Hilden, Germany). Single-end 75bp reads were generated by the NextSeq (Illumina, San Diego, CA) located at the Scripps Next Generation Sequencing Facility. Data processing steps were performed using the nf-core rnaseq version 3.6 pipeline using the GRCh38 reference genome. The pipeline can be found here: https://doi.org/10.5281/zenodo.6327553.

### Chromatin immunoprecipitation

Cells were crosslinked with 1% formaldehyde directly added to tissue culture media and lysed in 5 mM PIPES (pH 8.0), 85 mM KCL, and 0.5% NP40, and cell nuclei were recovered. Nuclei were lysed in 1% SDS, 10 mM EDTA (pH 8.0), and 50 mM Tris-HCL (pH 8.0). Nuclei were sonicated to a DNA average size of 300bp. ChIP was performed as described (Herman et al., 2006) with Sirt6 antibody from Abcam (ab191385; Cambridge, UK). Data processing steps were performed using the nf-core chipseq version 1.2.2 pipeline using the GRCh38 reference genome. The pipeline can be found here: https://doi.org/10.5281/zenodo.4711243.

### Statistics

Data analysis was performed using STATA SE/17 software and Prism9. Linear regression models were used to examine the effect of single SNPs on specific outcome measures, accounting for GAA1 length, sex, and age. The outcome measures examined included quantitative neurological function (FARS/mFARS, visual acuity, T25FW, 9HPT, Z2 composite, and Z3 composite). Fisher’s exact test was used to test the effect of the SIRT6 genotype on the presence of cardiomyopathy, scoliosis, and diabetes. Parametric and non-parametric tests, comparing means and medians and accounting for repeated measures were appropriate, were used to compare variables (mFARS, visual acuity, age, sex, GAA1&2, age of onset, T25FW, 9HPT, and Z2 composite) between CT and TT (and TT_mod_).

## Data Availability

Data sets used for RNA-seq and ChIP-seq analysis on iPS-derived neurons are deposited in the GEO database under accession number GSE200907.
