# Supplementary material for: A non-synonymous single nucleotide polymorphism in SIRT6 predicts neurological severity in Friedreich ataxia
Source: Front Mol Biosci. 2022 Sep 5;9:933788. doi: 10.3389/fmolb.2022.933788 (PMC9483148; doi:10.3389/fmolb.2022.933788)
Supplement: Supplementary file 1 [file DataSheet1.PDF]

SUPPLEMENTAL DATA

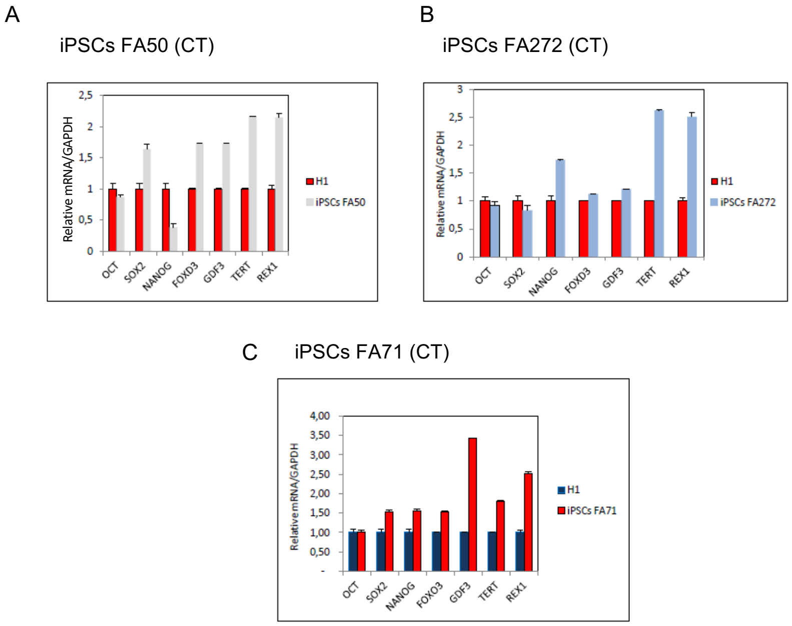

Supplemental figure 1. Analysis of pluripotency gene expression in three iPSC lines derived from FRDA patient fibroblasts harboring SIRT6 CT variant: (A) FA50, (B) FA272 (C) FA71, by quantitative real time PCR. The iPSC lines show similar or higher expression of the indicated pluripotency mRNAs compared to H1 hESCs. mRNA levels are normalized to GAPDH. Error bars indicate SEM of triplicate measurements.

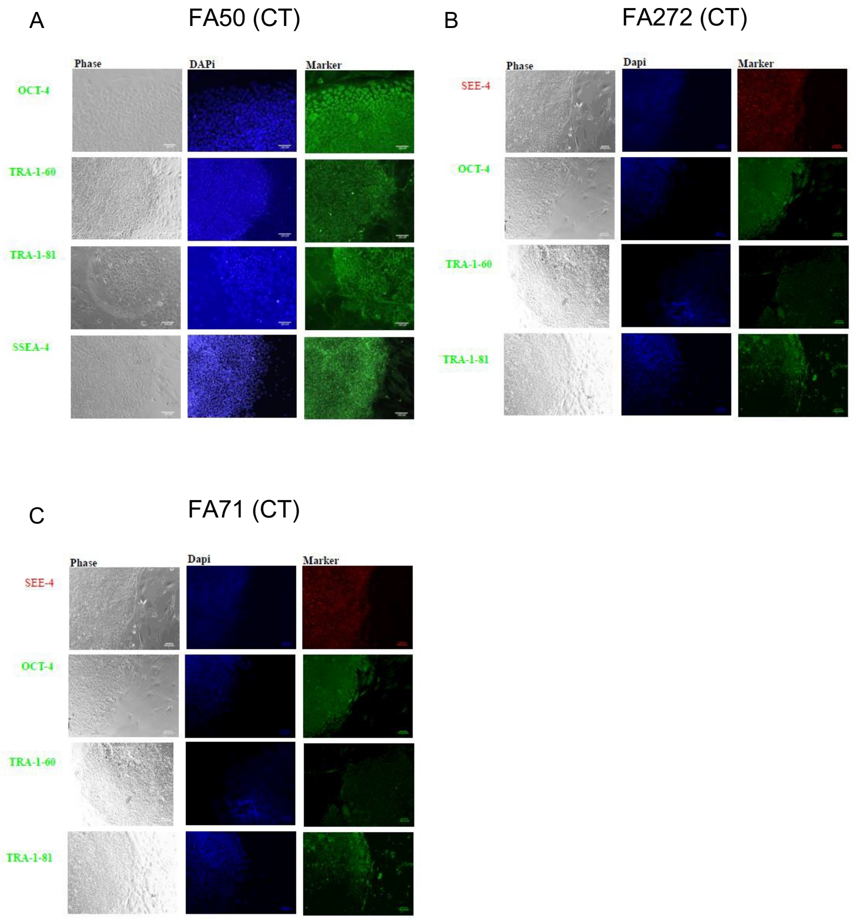

Supplemental figure 2. Immunostaining for pluripotency markers in (A) FA50, (B) FA272, and (C) FA71 iPSCs. Phase contrast (gray); nuclear staining (blue); pluripotency markers staining (green or red), as denoted by the colored text labels. Tra1-60 and Tra1-81, surface markers; SSEA-4, stage-specific embryonic antigens; Oct4, transcription factor. Scale bars represent 100 mm.

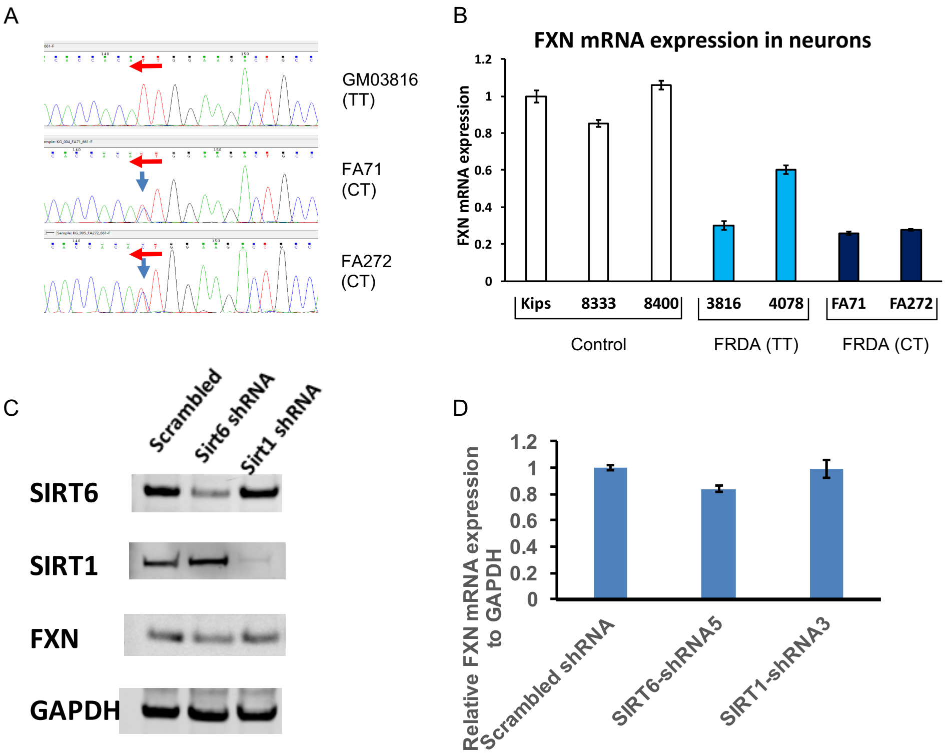

Supplemental Figure 3. (A) DNA sequence analysis of PCR products from the SIRT6 gene in FRDA iPSC-derived neuronal cells. **(B)** qRT-PCR quantification of *FXN* mRNA in iPSC-derived neuronal cells from control and FRDA patients. Repeat numbers and *SIRT6* SNP status are provided in Supplementary Table1. *FXN* mRNA levels are normalized to *GAPDH* mRNA and set to 1.0 for control Kips neurons. (C) Western blot confirmation of shRNA silencing of SIRT1 or SIRT6. (D) *FXN* mRNA expression with shRNA knockdown of SIRT1 or SIRT6 in FRDA iPSC-derived neurons.

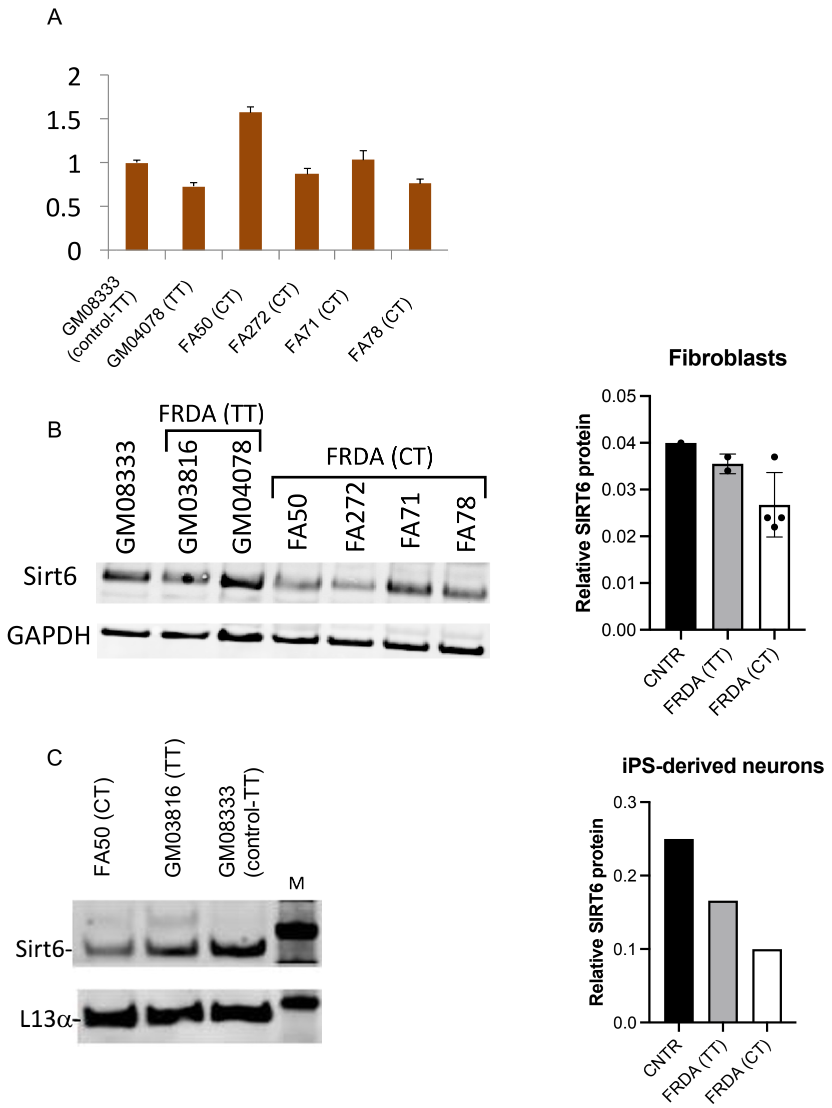

Supplemental Figure 4. *SIRT6* transcript and protein levels in fibroblasts from patients with CT *SIRT6*, patients with TT *SIRT6*, and one unaffected individual, as measured by (A) qRT-PCR and (B) western blotting with quantification (stats: Mann-Whitney test, p=n.s.). (C) Western blot and quantification of SIRT6 protein in iPSC-derived neurons from a healthy subject (GM08333; TT) and FRDA patients, FA50 (CT) and GM03816 (TT).

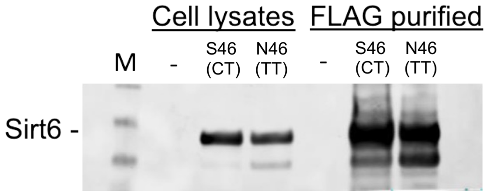

Supplemental Figure 5. Western blots for SIRT6 protein in HEK293 cells transfected with FLAG-tagged mammalian expression vectors encoding SIRT6 S46 or N46."-" denotes pcDNA 3.1 empty vector; pcDNA3.1 S46 expressing the rare Ser46 version and pcDNA3.1 N46 expressing the prevalent Asn46 version, for both total cell lysates and FLAG-purified protein.

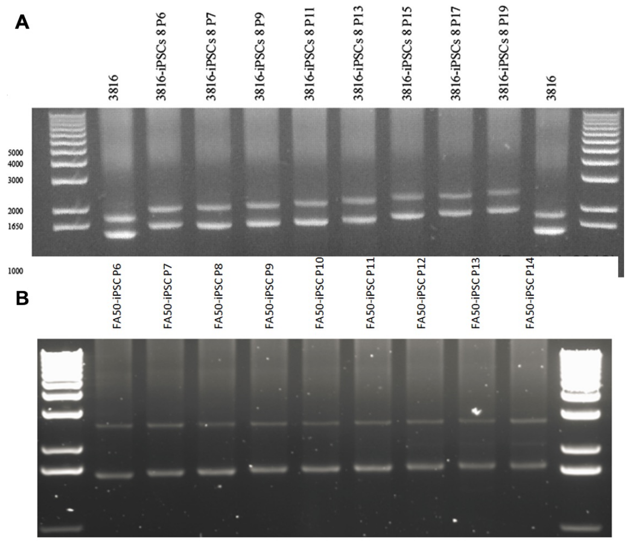

Supplemental Figure 6. GAA repeat expansion in FRDA iPSC lines (A) GM3816 (TT) and (B) FA50 (CT). iPSC lines were propagated for the indicated numbers of passages (P) and a portion of the *FXN* gene harboring the repeats was amplified by PCR and analyzed by agarose gel electrophoresis and ethidium staining of the PCR products, as in Ku et al. (2010) and Du et al. (2012). PCT products from the corresponding GM03816 fibroblast line are shown (indicated 3816). DNA length markers were run at the sides of each gel.

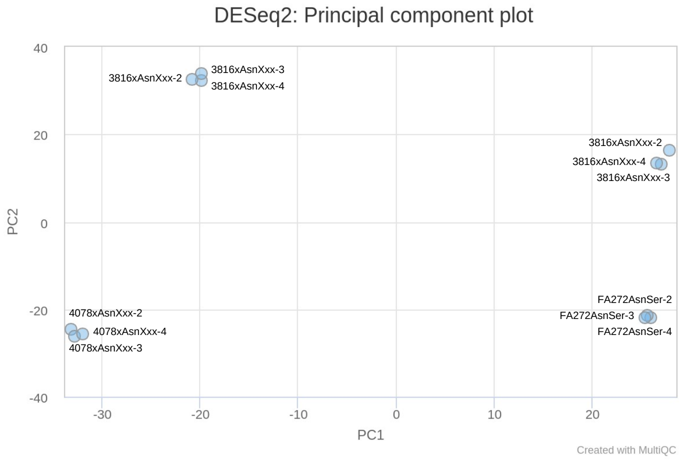

Supplemental figure 7. Principal component analysis of RNA-seq data. Samples were found to cluster according to both SIRT6 genotype (CT:FA71 and FA272, TT: GM04078 and GM03816) and GAA repeat lengths (FA71=229/1043; FA272= 566/866; GM04078= 420/541; GM03816=330/380).

SNP	rs34162626 SIRT5	rs1045288 SIRT3/PSMD13	rs228757 HDAC5	rs352493 SIRT6	rs34402301 HDAC10
Allele Frequency (%A)	0.961	0.263	0.259	0.058	0.945
9HPT (R^2, p value)	0.3 0.36	0.29, 0.63	0.30, 0.47	0.31, 0.223	0.32, 0.065
T25FW (R^2, p value)	0.36, 0.14	0.34, 0.94	0.36, 0.25	**0.40, 0.007**	**0.39, 0.039**
LCLA (R^2, p value)	0.20, 0.11	0.14, 0.24	0.13, 0.51	0.14, 0.34	0.12, 0.78
Z3 (R^2, p value)	0.39, 0.029	0.35, 0.75	0.37, 0.24	**0.39, 0.035**	0.38, 0.092
FARS (R^2, p value)	0.34, 0.48	0.34, 0.9	0.37, 0.70	**0.36, 0.078**	0.34, 0.28
Scoliosis (R^2, p value)	0.23, 0.95	0.28, 0.07	0.24, 0.42	0.24, 0.68	0.26, n.s.
Cardiomyopathy (R^2, p value)	0.45, 0.44	0.46, 0.097	0.45, 0.26	0.48, 0.13	0.44, 0.55

Supplemental table 1. Associations of HDAC gene polymorphisms with parameters of FRDA disease progression in 88 individuals with FRDA. Values with $p<0.05$ are in bold. SNP=single nucleotide polymorphism, 9HPT=9-hold peg test, T25FW=timed 25-foot walk, LCLA=low-contrast letter acuity, FARS=Friedreich ataxia rating scale. Z score calculated as (value-mean)/standard deviation, and Z3 is the sum of the Z-scores for 9HPT, T25FW, and LCLA.

Pathway name	# genes (DE/all)	p value
Bacterial invasion of epithelial cells	**19**/39	7.313e-5
Phospholipase D signaling pathway	**23**/59	1.690e-4
Rap1 signaling pathway	**35**/97	2.314e-4
Tight junction	**27**/75	0.001
Platelet activation	**21**/54	0.001
Viral Carcinogenesis	**29**/85	0.002
Leukocyte transendothelial migration	**22**/55	0.003
Prion disease	**23**/108	0.006
Focal anhesion	**32**/103	0.010
Platinum drug resistance	**13**/38	0.011

Supplemental table 2. Top enriched pathways for DE genes in FRDA patients with CT vs. TT *SIRT6*, identified within the KEGG Pathway Database (Release 96.0+/11-21, Nov 20).

Gene symbol	Gene name	p value	logFC	FC (TT Vs. CT)	Function	Pathway(s)
RETN	resistin	0.0327	0.1330	1.0966=	Hormone that seems to suppress insulin ability to stimulate glucose uptake into adipose cells	adipogenesis
SEMA3E	semaphorin 3E	0.0370	-0.2590	0.8357	important in cell-cell adhesion and cytoskeleton organization and vascular development. Also plays a role in ensuring the specificity of synapse formation	brain development
SLC25A45	solute carrier family 25 member 45	0.0063	-0.0857	0.9423	solute carrier	cell volume and ion homeostasis
SLC12A9	solute carrier family 12 member 9	0.0297	-0.1370	0.9094	solute carrier	
FLVCR1	feline leukemia virus subgroup C cellular receptor 1	0.0010	-0.0977	0.9345	Heme transporter that exports cytoplasmic heme.	cellular iron homeostasis
PHF21A	PHD finger protein 21A	0.0260	-0.1280	0.9151	Component of the BHC complex, a corepressor complex that represses transcription of neuron-specific genes in non-neuronal cells.	chromatin organization
CREB1	cAMP responsive element binding protein 1	0.0461	-0.1940	0.8742	Stimulates transcription upon binding to the DNA cAMP response element (CRE). Involved in the synchronization of circadian rhythmicity and the differentiation of adipose cells.	circadian rhythm
NFIL3	nuclear factor, interleukin 3 regulated	0.0270	-0.1600	0.8950	regulation of circadian rythym. Represses transcription from promoters with ATF sites.	
PRDX5	peroxiredoxin 5	0.0036	0.1910	1.1416	cytoprotective antioxidant	detoxification of reactive oxygen species
NOTCH2	notch 2	0.0281	-0.0855	0.9425	receptor for membrane bound ligands. Upon activation, it forms a transcriptional activator complex.	development; cell fate determination
TDP1	tyrosyl-DNA phosphodiesterase 1	0.0464	-0.2380	0.8479	DNA repair enzyme	DNA damage repair
HIST1H2BD	histone cluster 1, H2bd	0.0134	-0.2110	0.8639	cellular senescence and mitotic prophase pathways	
FANCA	Fanconi anemia complementation group A	0.0042	-0.0977	0.9345	DNA repair protein	
CDKN2D	cyclin dependent kinase inhibitor 2D	0.0096	-0.0826	0.9444	cell cycle regulator	
AKR1C3	aldo-keto reductase family 1, member C3	0.0114	0.2010	1.1495	catalyzes the NADH/NADPH dependent reduction of ketosteroids to hydroxysteroids. SIRT6 target	metabolism
MARCKS	myristoylated alanine rich protein kinase C substrate	0.0263	-0.2010	0.8699	the most prominent cellular substrate for protein kinase C. This protein binds calmodulin, actin, and synapsin. MARCKS is a filamentous (F) actin cross-linking protein	
PLPP2	phospholipid phosphatase 2	0.0307	-0.0995	0.9334	Magnesium-independent phospholipid phosphatase that catalyzes the dephosphorylation of a variety of glycerolipid and sphingolipid phosphate esters	
ATP5O	ATP synthase, H+ transporting, mitochondrial F1 complex, O subunit	0.0252	0.1520	1.1111	Produces ATP from ADP in the presence of a proton gradient across the membrane which is generated by electron transport complexes of the respiratory chain	metabolism; ATP synthesis
PGS1	phosphatidylglycerophosphate synthase 1	0.0414	-0.1390	0.9081	Functions in the biosynthesis of the anionic phospholipids phosphatidylglycerol and cardiolipin	metabolism; cardiolipin biosynthesis

CHCHD2	coiled-coil-helix-coiled-coil-helix domain containing 2	0.0045	0.1670	1.1227	Transcription factor that binds to O2 responsive element of COX4I2 and activates its transcription under hypoxic or normoxic conditions	mitochondrial protein import
LIN7A	lin-7 homolog A, crumbs cell polarity complex component	0.0211	-0.0888	0.9403	Plays a role in establishing and maintaining the asymmetric distribution of channels and receptors at the plasma membrane of polarized cells.	neurotransmitter and synapse regulation
ADM	adrenomedullin	0.0204	-0.2860	0.8202	vasodilator peptide hormone known to increase tolerance of cells to oxidative stress	response to hypoxia
HIF3A	hypoxia inducible factor 3 alpha subunit	0.0055	-0.0886	0.9404	Acts as a transcriptional regulator in adaptive response to low oxygen tension	
SDR16C5	short chain dehydrogenase/reductase family 16C, member 5	0.0207	-0.0823	0.9446	oxidation of retinol to retinaldehyde	retinol metabolic processes

Supplementary table 3. Top differentially expressed genes in blood from FRDA patients with TT Vs. CT *SIRT6*. Gene expression data analyzed with GEO2R software package.
